# Olmsted syndrome: exploration of the immunological phenotype

**DOI:** 10.1186/1750-1172-8-79

**Published:** 2013-05-21

**Authors:** Dina Danso-Abeam, Jianguo Zhang, James Dooley, Kim A Staats, Lien Van Eyck, Thomas Van Brussel, Shari Zaman, Esther Hauben, Marc Van de Velde, Marie-Anne Morren, Marleen Renard, Christel Van Geet, Heidi Schaballie, Diether Lambrechts, Jinsheng Tao, Dean Franckaert, Stephanie Humblet-Baron, Isabelle Meyts, Adrian Liston

**Affiliations:** 1 Autoimmune Genetics Laboratory, VIB, Leuven, Belgium; 2 Department of Microbiology and Immunology, University of Leuven, 3000, Leuven, Belgium; 3 T-Life Research Center, Fudan University, Shanghai, China; 4 BGI-Shenzhen, Shenzhen, China; 5 Laboratory of Translational Genetics, Vesalius Research Center, VIB and University of Leuven, Leuven, Belgium; 6 Pathology, Faculty of Medicine, University Hospital Leuven, Leuven, Belgium; 7 Anesthesiology, Faculty of Medicine, University Hospital Leuven, Leuven, Belgium; 8 Dermatology, Faculty of Medicine, University Hospital Leuven, Leuven, Belgium; 9 Pediatric Hemato-Oncology, Faculty of Medicine, University Hospital Leuven, Leuven, Belgium; 10 Pediatrics, Faculty of Medicine, University Hospital Leuven, Leuven, Belgium

**Keywords:** Olmsted syndrome, TRPV3, IgE, Eosinophil, Follicular T cell

## Abstract

**Background:**

Olmsted syndrome is a rare congenital skin disorder presenting with periorifical hyperkeratotic lesions and mutilating palmoplantar keratoderma, which is often associated with infections of the keratotic area. A recent study identified de novo mutations causing constitutive activation of TRPV3 as a cause of the keratotic manifestations of Olmsted syndrome.

**Methods:**

Genetic, clinical and immunological profiling was performed on a case study patient with the clinical diagnosis of Olmsted syndrome.

**Results:**

The patient was found to harbour a previously undescribed 1718G-C transversion in *TRPV3*, causing a G573A point mutation. In depth clinical and immunological analysis found multiple indicators of immune dysregulation, including frequent dermal infections, inflammatory infiltrate in the affected skin, hyper IgE production and elevated follicular T cells and eosinophils in the peripheral blood.

**Conclusions:**

These results provide the first comprehensive assessment of the immunological features of Olmsted syndrome. The systemic phenotype of hyper IgE and persistent eosinophilia suggest a primary or secondary role of immunological processes in the pathogenesis of Olmsted syndrome, and have important clinical consequences with regard to the treatment of Olmsted syndrome patients.

## Background

Olmsted syndrome was first described in 1927 as a combination of hyperkeratotic lesions and palmoplantar keratoderma severe enough to result in spontaneous digit amputation [1]. A recent study identified *de novo* gain-of-function mutations in the thermosensitive cation channel *TRPV3*, most frequently Gly573Ser, as causative in 6 OS patients [2]. The 573 residue is also mutated in two autosomal dominant rodent models of OS, DS-Nh mice and WBN/Kob-Ht rats, both of which develop hyperkeratosis [3]. By contrast, Trpv3-deficient mice display mild disorganisation of the epidermis, but no hyperkeratosis [4,5], indicating that gain-of-function is required for disease. The mechanism by which increased activity of TRPV3 leads to pathogenesis is unknown. Expression of TRPV3 in keratinocytes and *in vitro* studies suggest that elevated keratinocyte apoptosis could drive disease [2], however it should be noted that TRPV3 is also expressed by Langerhans dendritic cells in the skin [6], and thus an immunological origin for disease cannot be excluded. A review of published cases noted the common concordance of recurrent bacterial and candida infections in keratotic areas [7], supporting a link between dermal and immunological defects which has not been further explored.

## Methods

### Human subjects

The study was approved by the Ethics Committee of UZ Leuven and informed consent was obtained from the patient and his parents. Healthy volunteers for immune phenotyping were 3 males and 8 females, including the healthy sister of the patient, and were all Caucasians between the ages of 21 and 24 years. Blood was drawn from each donor only after informed consent had been given. All healthy controls used were matched for ethnicity to the patient family (self-declared Flemish ethnicity for at least 3 generations).

### Genetic analysis

DNA was isolated from heparinized blood of the patient and all-exome sequencing was performed as described earlier [8]. Detected variants were filtered to remove those which were non-exonic, synonymous or non-rare (allele frequency greater than 0.5% in dbSNP, 1000 Genomes or HapMap, or present in YH). Confirmation and frequency testing of the *TRPV3* 1718G-C transition was performed via Sequenom in a blinded manner using iPLEX technology on a MALDI-TOF based MassARRAY Compact Analyser (Sequenom Inc., CA, USA) as described previously [9]. Prediction of mutations on protein function were performed using Polyphen-2 (v2.2.2r398) [10] and SIFT [11].

### Flow cytometry

Peripheral blood mononuclear cells (PBMC) were isolated from heparinized blood of patients and controls using lymphocyte separation medium (LSM, MP Biomedicals) and frozen in 10% DMSO (Dimethyl sulfoxide, Sigma). Thawed cells were stained with eBioscience antibodies against CD11c (3.9), CD3 (SK7), CD4 (RPA-T4), CD8a (RPA-T8), CD19 (HIB19), CD45Ra (HI100), CD56 (MEM188), HLA-DR (LN3), FOXP3 (206D, Biolegend), IFN-γ (4S.B3 IL-17 (eBio64DEC17), IL-2 (MQ1-17H12), CXCR5 (IgG23, R&D), CD31 (WM-59), CCR7 (3D12), IgM (MHM-88, Biolegend), CD27 (O323), IgE (IgE21), CD24 (eBioSN3, SN3 A5-2H10), CD38 (HIT2), γδTCR (B1.1), Vα24Jα18 (6B11), CD56 (MEM188), CD14 (61D3), CD123 (6H6) and IL-4 (8D4-8). For cytokine staining, T cells were stimulated *ex vivo* for five hours in 50 ng/ml PMA (Phorbol 12-myristate 13-acetate, Sigma) and 500 ng/ml ionomycin (Sigma) in the presence of GolgiStop (BD Biosciences) before staining. Prior to intracellular staining, cells were first surface stained as described, fixed and permeabilised using fixation/permeabilisation buffer (eBioscience) for Foxp3 staining or Cytofix/cytoperm (BD) for other intracellular stainings. All data were acquired on BD FACSCantoII and analysed with FlowJo (Tree star).

## Results

### Clinical features of index patient

**Figure 1:**
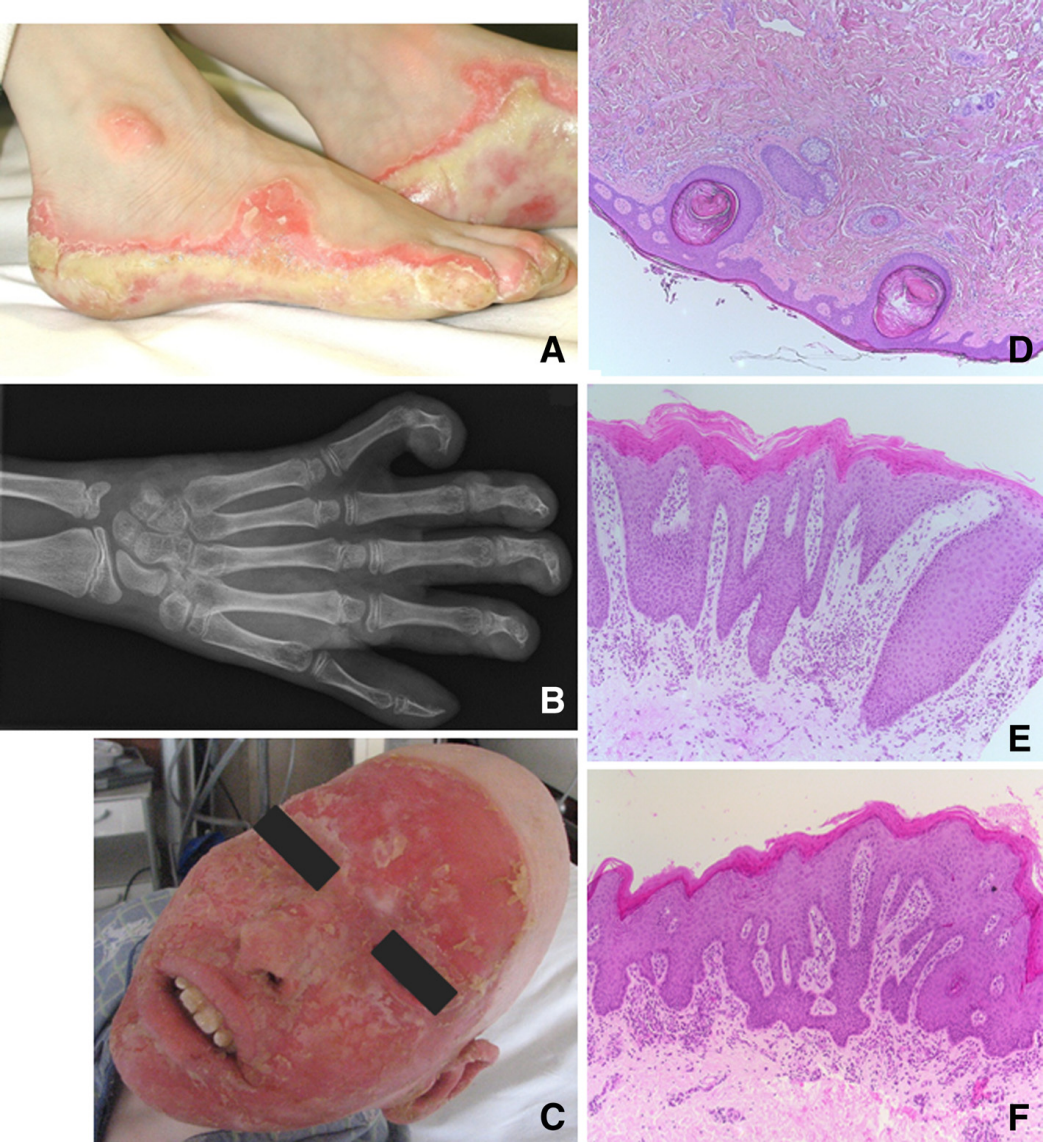
**Dermatological presentation of Olmsted syndrome case.** Physical examination revealed hyperkeratotic inflamed periorifical lesions on the face, foot soles, hands and genitals. Growth and development of the patient were severely impaired, with height and weight 4.3 and 6.2 standard deviations below normal, without the onset of puberty. **A)** Thick and macerated keratoderma on the feet together with onychodystrophy at all digits. **B)** X-ray of right hand, showing bone resorption and flexion contracture, corresponding with severe impairment of fine and gross motor skills. **C)** Facial erythematous with keratotic plaques ending sharply at the scalp, together with alopecia totalis, including eyebrows and eye lashes. **D)** Punch biopsy of scalp (H&E stain, 50×), showing epidermis of normal thickness and orthokeratosis. Hair follicles are rare and present with dilated infundibula and keratin plug. Inflammation and fibrosis is absent. **E)** Punch biopsy of affected skin (H&E stain,100×), showing confluent parakeratosis of the epidermis and elongation of the rete ridges. Moderate infiltration of mononuclear cells in the superficial dermis and normal deep dermis. **F)** Affected skin fragment (H&E stain, 100×), with prominent parakeratosis and acanthosis of the epidermis. The papillary dermis contains a moderate mononuclear infiltration.

The index patient is an 18 year old Caucasian male with Olmsted Syndrome, the second child of non-consanguineous, healthy parents. The boy was born at 31 weeks gestation and his neonatal course was complicated by intraventricular haemorrhage and neonatal convulsions. His skin condition was present from birth with erythematosquamous periorifical lesions on the face, progressively extending to involve feet, hands and genitals (Figure 1). Notably, initiation of skin lesions on the feet coincided with the patient starting to walk, and an injury on the thigh resulted in chronic lesions, indicating responsiveness of dermatological features to the environment. Initially the differential diagnosis of congenital pityriasis rubra pilaris, acrodermatitis enteropathica and chronic mucocutaneous candidiasis was put forward, however histologic examination was suggestive of psoriasis (Figure 1). The phenotypic diagnosis of Olmsted Syndrome was made at age 4 years. Skin lesions remain severe with chronic refractory itching and pain resulting in insomnia despite trials with topical and systemic corticosteroids, isotretinoin, vitamin D, topical and systemic calcineurin inhibitors, methotrexate, several antihistaminics, amitriptyline and morphine substrates. From the age of 8 years scrapings of the skin lesions were performed every 2 weeks under general anaesthesia to reduce contractures, purulent collections and fissures. Self-reported quality of life is still poor.

### Identification of a novel mutation in TRPV3

Genetic assessment of the case was performed via exome sequencing. No mutations were observed in candidate genes combining immunological and dermatological manifestations, including *FOXP3*, *SPINK5* and *FLG*. Additionally, no mutations in *MBTPS2* (recently shown to cause Olmsted syndrome) [12] were observed. Filtering out common polymorphisms identified a novel heterozygous G1718C transversion in *TRPV3*, resulting in a Gly573Ala missense mutation. The G1718C mutation was confirmed by Sequenom and was absent in available healthy family members and 871 healthy controls. While *de novo* mutation fits the available genetic and clinical familial data, DNA from the father was not available to exclude paternal inheritance. The Gly573Ala mutation has not been previously described in Olmsted Syndrome, however mutations in this amino acid location (Gly573) cause disease in both patients and rodent models [2,3,13]. The Gly573 location is predicted to be highly sensitive to mutation, with all possible amino acid changes, including Gly573Ala, predicted to be damaging (Table 1), providing the genetic diagnosis of Olmsted syndrome.

**Table 1:** Predicted effect of TRPV3 mutations

**Mutation**	**Phenotype**	**Polyphen-2**	**SIFT**
G573A	Olmsted (this study)	0.775 (sensitivity: 0.85, specificity: 0.92)	0.01
G573C	Olmsted [2]	0.995 (sensitivity: 0.68, specificity: 0.97)	0.00
G573D	Unknown	0.924 (sensitivity: 0.81, specificity: 0.94)	0.00
G573E	Unknown	0.941 (sensitivity: 0.80, specificity: 0.94)	0.00
G573F	Unknown	0.996 (sensitivity: 0.55, specificity: 0.98)	0.00
G573	Native allele	NA	NA
G573H	Unknown	0.996 (sensitivity: 0.55, specificity: 0.98)	0.00
G573I	Unknown	0.992 (sensitivity: 0.70, specificity: 0.97)	0.00
G573K	Unknown	0.941 (sensitivity: 0.80, specificity: 0.94)	0.00
G573L	Unknown	0.970 (sensitivity: 0.77, specificity: 0.96)	0.00
G573M	Unknown	0.999 (sensitivity: 0.14, specificity: 0.99)	0.00
G573N	Unknown	0.941 (sensitivity: 0.80, specificity: 0.94)	0.00
G573P	Unknown	0.970 (sensitivity: 0.77, specificity: 0.96)	0.00
G573Q	Unknown	0.992 (sensitivity: 0.70, specificity: 0.97)	0.00
G573R	Unknown	0.960 (sensitivity: 0.78, specificity: 0.95)	0.00
G573S	Olmsted [2,13]	0.048 (sensitivity: 0.94, specificity: 0.83)	0.05
G573T	Unknown	0.941 (sensitivity: 0.80, specificity: 0.94)	0.00
G573V	Unknown	0.960 (sensitivity: 0.78, specificity: 0.95)	0.00
G573W	Unknown	0.999 (sensitivity: 0.14, specificity: 0.99)	0.00
G573Y	Unknown	0.996 (sensitivity: 0.55, specificity: 0.98)	0.00
W692G	Olmsted [2]	0.999 (sensitivity: 0.14, specificity: 0.99)	0.00

### Clinical and diagnostic manifestations of immune dysregulation in the index patient

Within 3 months of birth the patient demonstrated severe immunological components of the disease, with frequent bacterial and fungal infections of the skin (especially *Candida albicans*) requiring chronic treatment with antibiotics and antifungals. Invasive bacterial or fungal infections were never observed. Analysis of antibody isotypes indicated normal IgM and IgG concentrations, although IgG3 was frequently at or below the lower end of the normal range (from the age of 3 years on) and IgA was consistently elevated (Table 2). Antibody isotypes were first measured at the age of 3 years, with the exception of IgE, which was not measured until 17 years, when hyper IgE with values well above the normal range were observed. The patient’s NIH score for HyperIgE syndrome is 34, with values >40 being suggestive of AD HyperIgE syndrome. No autoantibodies were detected (data not shown).

**Table 2:** Immunoglobulin measurement from Olmsted syndrome case at 17 years of age

**Isotype**	**Blood concentration (g/L)**	**Normal range**
IgG	10.6	5.76-12.65
→IgG2	2.77	1.06-6.10
→IgG3	0.19	0.18-1.63
IgA	3.33	0.18-2.32
IgM	1.45	0.30-1.59
IgE	4241 kU/L	< 35 kU/L

### Elevated follicular T cells and systemic eosinophilia in the index patient

**Figure 2:**
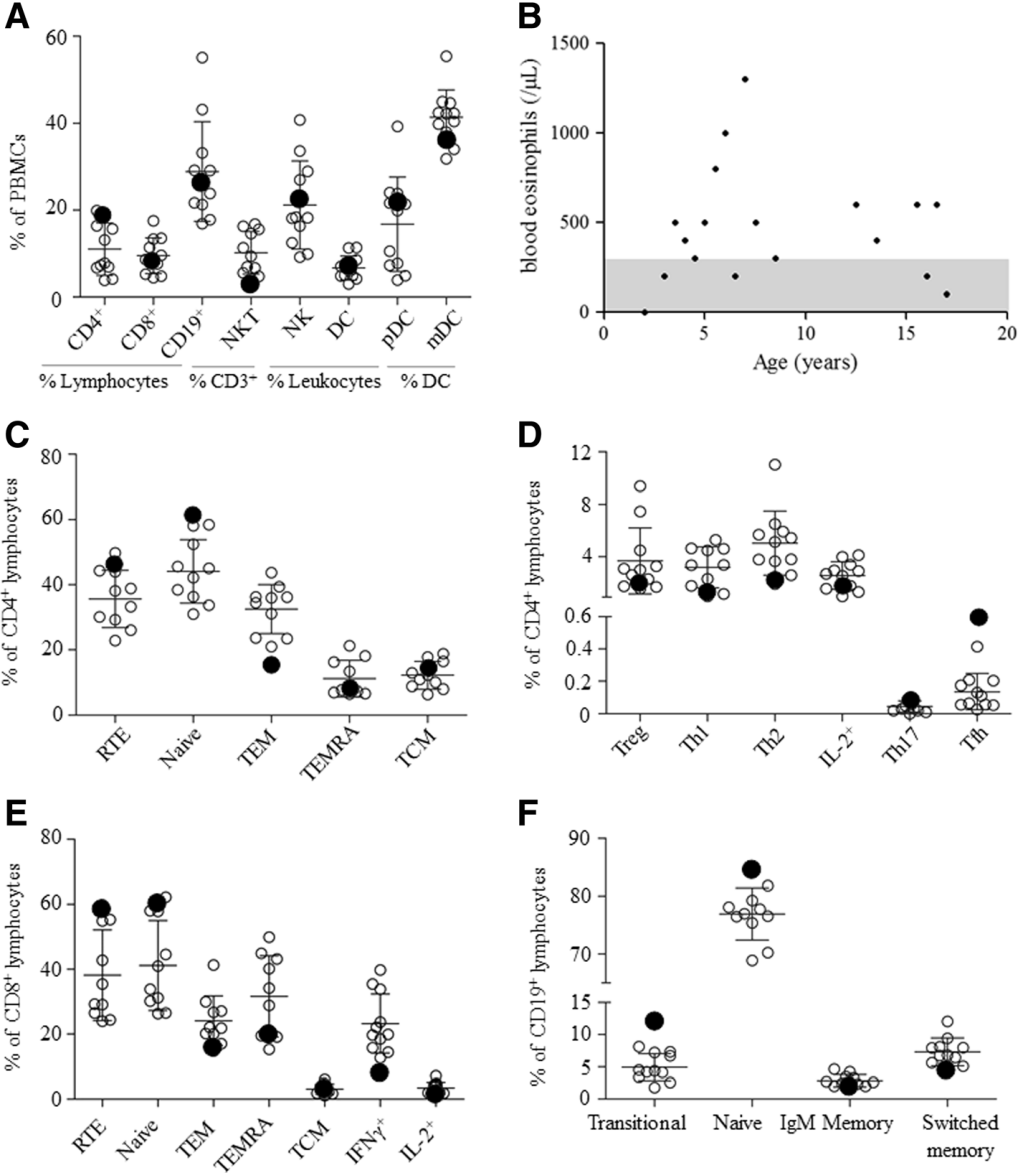
**Immunological profile of an Olmsted syndrome patient.** Relative frequencies of peripheral blood leukocyte populations at 18 years of age, unless otherwise indicated. **A)** Major blood leukocyte subsets. **B)** Fluctuating peripheral blood eosinophil numbers over time, grey zone indicates normal range. **C)** Major CD4 T lymphocyte subsets. **D)** Helper T cell lineages. **E)** CD8 T lymphocyte subsets. **F)** B cell subsets. The described Olmsted Syndrome case is indicated by a filled circle and healthy age-matched controls are indicated as empty circles. The mean and standard deviation (error bars) shown exclude the values for the patient. TCM, central memory T cell; TEM, effector memory T cell; TEMRA, CD45RA-expressing effector memory T cell; Tfh, follicular T cell; Th1, type 1 helper T cell; Th2, type 2 helper T cell; Th17, IL-17-expressing helper T cell; RTE, recent thymic emigrant.

In order to account for the immunological components of the case, we profiled the peripheral immune system. Of the major mononuclear leukocyte cell types surveyed the patient values were within one standard deviation of the mean of the healthy controls, with the exception of NKT cells (Figure 2A and Table 3). Granulocyte numbers/function/chemotaxis were normal, except for a chronic elevation in eosinophils, which frequently reached levels consistent with eosinophilia (Figure 2B). Within the T lymphocyte population we found no sign of excessive T cell activation, despite the history of chronic infection (Figure 2C-E and Table 4). Indeed, naïve cells were over-represented within both the CD4 and CD8 T cell populations, while activated and effector memory T cells subsets were as low, or lower, than healthy controls. T cell proliferation tests with candida, tetanus, PHA and PWM were normal (data not shown). The one effector T cell population that was elevated in the OS patient was the follicular helper (Tfh) CD4 T cell, which was 4-fold more abundant. Despite the function of circulating Tfh cells in promoting B cell responses [14], analysis of B cell subsets revealed no systemic activation, with transitional and naïve B cells at or above the high end of the healthy range, and memory and switched B cells at the low end (Figure 2F and Table 5). IgE^+^ B cells were not increased in circulation, indicating that the presence of IgE-secreting plasma cells may be limited to secondary lymphoid sites.

**Table 3:** Frequency of major leukocyte subsets from patient with mutation in TRPV3 and age-matched healthy controls

**Subset**	**Defining surface markers**	**Patient (%)**	**Healthy volunteers (%)**	
			**Mean (SD)**	**Median (min-max)**
T cells	CD3^+^	25.0	21.7 (10.7)	20.2 (8.48-41.6)
B cells	CD19^+^	25.7	28.9 (11.5)	26.5 (16.9-55.1)
γδ T cells	γδ TCR^+^	0.25	0.23 (0.21)	0.15 (0.04-0.72)
iNKT cells	CD3^+^Vα24Jα18^+^	0.49	0.52 (0.25)	0.55 (0.09-0.89)
NKT cells	CD3^+^CD56^+^	2.49	10.2 (4.91)	9.35 (4.15-16.8)
NK cells	CD3^-^CD19^-^CD14^-^CD11c^-^CD56^+^	22.0	21.2 (10.1)	18.2 (9.17-40.8
Dendritic cells	CD3^-^CD19^-^CD14^-^CD56^-^	6.67	6.66 (2.76)	6.21 (2.96-11.4)
→mDC	CD11c^+^HLA-DR^+^	35.5	41.4 (6.17)	42.2 (31.8-55.4)
→pDC	CD11c^-^CD123^+^	21.3	16.8 (10.9)	19.9 (3.88-39.25)

T cells are shown as percentage total leukocytes; B cells and γδ T cells are shown as percentage total lymphocytes; iNKT and NKT cells as percentage T cells; NK cells and dendritic cells are shown as percentage total leukocytes while mDC and pDC are shown as percentage dendritic cells.

**Table 4:** T lymphocyte profile of Olmsted syndrome case and age-matched healthy controls

**Subset**	**Defining surface markers**	**Patient (%)**	**Healthy volunteers (%)**	
			**Mean (SD)**	**Median (min-max)**
T cells	CD3^+^	25.0	21.7 (10.7)	20.2 (8.48-41.6)
***CD4***^***+***^***subsets***				
CD4^+^ T cells	CD4^+^CD8^-^	18.2	11.0 (6.03)	7.79 (3.82-19.9)
→Treg	Foxp3^+^	1.91	3.67 (2.52)	2.97 (1.54-9.37)
→Th1	IFNγ^+^	1.22	3.18 (1.57)	3.36 (0.96-5.28)
→Th2	IL-4^+^	2.14	5.03 (2.45)	5.12 (1.90-11.0)
→Th17	IL-17^+^	0.08	0.05 (0.04)	0.03 (0–0.1)
→IL-2^+^	IL-2^+^	1.69	2.25 (1.06)	2.67 (0.95-4.11)
→Tfh	CD45RA^-^CXCR5^+^	0.59	0.14 (0.11)	0.11 (0.026-0.41)
→Naïve	CD45RA^+^CCR7^+^	61.3	44.1 (9.73)	43.6 (31.0-58.4)
→RTE	CD45RA^+^CCR7^+^CD31^+^	46.3	35.6 (8.77)	35.7 (22.8-49.7)
→TCM	CD45RA^-^CCR7^+^	14.6	12.2 (4.24)	11.6 (6.36-18.7)
→TEM	CD45RA^-^CCR7^-^	15.6	32.5 (7.54)	35.2 (21.0-43.7)
→TEMRA	CD45RA^+^CCR7^-^	8.50	11.9 (5.55)	7.78 (6.52-21.2)
***CD8***^***+***^***subsets***				
CD8^+^ T cells	CD4^-^CD8^+^	7.75	9.51 (4.12)	8.64 (4.45-17.5)
→IFNγ^+^	IFNγ^+^	8.1	23.3 (9.09)	19.9 (12.7-40.0)
→IL-2^+^	IL-2^+^	1.48	3.46 (1.74)	2.82 (1.78-7.17)
→Naïve	CD45RA^+^CCR7^+^	60.9	41.2 (13.9)	37.4 (26.3-62.2)
→RTE	CD45RA^+^CCR7^+^CD31^+^	59.0	38.2 (14.0)	32.3 (24.0-60.0)
→TCM	CD45RA^-^CCR7^+^	3.08	3.11 (1.52)	2.91 (1.08-6.00)
→TEM	CD45RA^-^CCR7^-^	16.0	24.1 (7.63)	22.0 (14.8-41.3)
→TEMRA	CD45RA^+^CCR7^-^	20.1	31.6 (12.5)	31.2 (15.3-49.9)

The frequency of CD3^+^ cells is shown as percentage from all leukocytes. CD4^+^ subsets and CD8^+^ subsets are from percentage CD4^+^ and CD8^+^ respectively.

**Table 5:** Relative proportion of B cell subsets in the Olmsted syndrome case and age-matched healthy controls

**Subset**	**Definition**	**Patient (%)**	**Healthy volunteers (%)**	
			**Mean (SD)**	**Median (min-max)**
B cells	CD19^+^	25.7	28.9 (11.5)	26.5 (16.9-55.1)
→Naïve	IgM^+^CD27^-^	84.5	76.9 (4.51)	76.9 (68.9-84.7)
→Memory	IgM^+^CD27^+^	1.91	2.84 (1.00)	2.58 (1.61-4.65)
→Switched memory	IgM^-^CD27^+^	4.40	7.30 (2.15)	6.82 (4.3-12.0)
→IgE^+^	IgE^+^	0.09	0.13 (0.09)	0.11 (0.04-0.37)
→Transitional	CD24^hi^CD38^hi^	12.0	4.94 (2.10)	4.63 (1.76-8.14)

CD19^+^ B cells are shown as percentage of total lymphocytes; all other B cell subsets are shown as percentage of CD19^+^ B cells.

## Discussion

Olmsted Syndrome is considered a dermatological disease based on the defining clinical features, however the frequency of cutaneous infections in patients indicates at least some immunological involvement. Here we have investigated the immunological profile of a patient confirmed to have Olmsted Syndrome by independent clinical and genetic diagnoses. The concordance in this patient of frequent cutaneous infections, psoriasis-like infiltration, hyper IgE, chronic eosinophilia and elevated follicular T cells in the peripheral blood all point to a novel immunological component of Olmsted Syndrome. While the penetrance of immunological manifestations in other cases of Olmsted Syndrome remains to be determined, these results have important pathophysiological implications. Based on dermatological-immunological interaction described here, two mechanistic models warrant further testing.

The first model of disease development supported by these observations is that of a disease process driven by primary defects in keratinocytes. It has been reported that constitutive activation of TRPV3 in keratinocytes drives apoptosis [2]. Either in response or in addition to this apoptosis, excessive keratin production drives the cutaneous changes. In this model, the link between the dermatological and immunological components would be mediated via barrier disturbance allowing frequent cutaneous infections, which in turn would cause secondary eosinophilia, Tfh polarisation and differentiation of IgE-secreting plasma cells. In support of this model is the known association of mutations in the keratin genes with other palmoplantar keratodermas [15], indicating that primary changes in keratinocytes can indeed drive the major dermatological features. In addition, elevated IgE and other immunological features are observed, albeit rarely, in primary dermatological diseases such as Netherton syndrome and Ichthyosis vulgaris [16]. Less supportive of this model is the mechanism by which increased keratinocyte apoptosis results in thickening, rather than thinning, of the skin, and why thickening would reduce the efficacy of the skin as an immunological barrier. Furthermore, the immunological features of this patient are not commonly observed in other cases of barrier disruption, although this result may simply be due to a lack of investigation into any immunological phenotype. Finally, the sharp barrier between affected and unaffected regions remains a curious and unexplained feature in a primary disease of keratinocytes.

Based on the case study presented here we propose a second, alternative model of Olmsted Syndrome development. While TRPV3 is described as having a keratinocyte-specific gene expression profile, the immunological gene express atlas also reports high expression of TRPV3 within Langerhans dendritic cells, uniquely among immunological cell types [6]. It is therefore possible that mutations causing constitutive activation of TRPV3 may also alter the properties of Langerhans cells by impeding dermatological tolerance and driving local autoimmunity. In this case, the characteristic dermatological features of the disease may arise as secondary effects, such as occurs in IPEX or APS-1 [16]. In support of this model is the effect of 17(R)-resolvin D1, a specific inhibitor for TRPV3 [17]. This inhibitor, produced as a leukocyte metabolite, has strong systemic anti-inflammatory properties independent of the skin barrier [18,19], indicating that TRPV3 does have primary immunological functions. In addition, the excessive inflammatory response to injury observed in this patient would be consistent with a primary immune disorder, and the sharp demarcation of affected areas is a common feature of immune-driven inflammation, such as psoriasis and Langerhans cell histiocytosis. Further research is needed to confirm the ubiquity of these findings and to test the two competing models of pathophysiology they inspire.

## Conclusions

Olmsted Syndrome is a rare congenital skin disorder, accompanied by frequent infections. In this report we describe the immunological phenotype of a patient with Olmsted Syndrome, confirmed by independent clinical and genetic diagnoses. We found a variety of immunological disturbances, including frequent dermal infections, hyper IgE levels, chronic eosinophilia and elevated follicular T cells in the peripheral blood. These results illuminate for the first time the immunological component of Olmsted Syndrome, and raise two competing hypotheses for disease mechanism: a primary dermatological disease which can cause secondary immunological dysregulation; or a primary cutaneous immunological disease, which manifests with strong dermatological effects. Investigation of additional cases and rodent models may aid in determining which of these disease mechanisms is at play in this devastating disease.

## Competing interests

The spouse of A. Liston is an employee of UCB Pharma. The other authors declare no conflict of interests.

## Authors’ contributions

DD-A, JD and DF performed immunoassays. JZ, KAS, LVE, TVB, DL, and JT performed genetic analysis. SZ, EH, MVdV, MM, CVG, HS, SH-B and IM provided clinical diagnostics and analysis. IM and AL designed the study and wrote the manuscript with the support of all authors. All authors read and approved the final manuscript.

## Authors’ information

Equal contribution first authors: Dina Danso-Abeam, Jianguo Zhang and James Dooley.

Equal contribution senior authors: Isabelle Meyts and Adrian Liston.
